# MOSAIC, an example of comprehensive and integrated social and health care: care and practices oriented towards personal recovery

**DOI:** 10.3389/frhs.2023.1174594

**Published:** 2023-08-04

**Authors:** Ivan Cano-Prieto, Gemma Prat-Vigué, Rut Vilanova-Masana, Aida Guillaume-Cornet, Rosa Giralt Palou, Gemma Lana-Francos, Salvador Simó-Algado

**Affiliations:** ^1^ Grup de Recerca en Innovació en Salut Mental i Benestar Emocional (ISaMBeS), Institut de Recerca i Innovació en Ciències de la Vida i de la Salut a la Catalunya Central (IRIS-CC), Vic, Spain; ^2^ Divisió de Salut Mental, Althaia, Xarxa Assistencial Universitària de Manresa, Manresa, Spain; ^3^ Facultat de Medicina. Universitat de Vic-Central de Catalunya (UVIC-UCC), Vic, Spain

**Keywords:** mental health recovery, personal recovery, integrated care, meaningful occupations, life project

## Abstract

**Background:**

The Mosaic project is a socio-health integration model that promotes the personal recovery of people with severe mental illness in a territory of Central Catalonia: the Bages region. The recovery approach in mental health care promotes meaningful activities and social inclusion for people with mental health disorders. The aim of this study is to examine the relationship between the level of meaningful activities and other factors associated with the mental health recovery model.

**Methods:**

A cross-sectional design was used. Participants (*n* = 59) signed an informed consent and completed the following standardized instruments: Engagement in Meaningful Activities Survey; The Connor-Davidson Resilience Scale; Hert Hope Scale; and Recovery Assessment Scale.

**Results:**

A Pearson correlation test was performed between the level of meaningful activities and life satisfaction, resilience, hope, and recovery. These data indicate that the amount of meaningful activities are strongly associated with variables related to the personal recovery process from mental health problems.

**Conclusions:**

The integration process of MOSAIC confirms the need to accompany the recovery processes through significant occupations.

## 1. Introduction

Social and health integration is a growing concern for governments, in a context of social and economic crises that demands efficiency (1–3).

Kodner and Spreeuwenberg defined the integration process as “a set of methods and models of financing, administration, organization, provision of services and clinical care designed to create connectivity, alignment and collaboration within and between the sector dedicated to caring [the social] and the sector dedicated to curing [health]” (4). Leutz (5) also emphasized this dimension of integrated care as a process, defining it as “the search to connect the health system”.

Integration processes between health and social services facilitate a continuum in the care of the population, focusing the intervention on the person’s needs (person-centered care). In addition, it demonstrates a special interest in accompanying the person from a paradigm of the social determinants of health (relationships between the environment, habits and routines, and personal health). Finally, a basic preventive and promotional care has been promoted (6).

### 1.1. Recovery model: a new approach to mental health

Personal recovery refers to the ways in which a person manages a mental health problem trying to restore or develop a meaningful life project, as well as a sense of belonging and a positive perception of identity that is independent of a mental health problem (7, 8). Recovery is a process of change by which individuals improve their health and well-being, lead their lives autonomously and strive to achieve their full potential. This approach has its origins in the historic “recovery movement”, which promoted, in the 1960s, the rights of people with mental health problems to receive decent therapeutic care as well as the consideration of the person with mental health problems as a competent individual that can make decisions about their life project and community functioning (9). At first, the initiatives focused on mental health laws, especially those that sanctioned involuntary and coercive interventions, but later changes were also proposed in the practice of mental health, especially from the appearance of new therapeutics, which would allow people with mental health problems to live in the community, and started the creation of rehabilitative resources in the community in order to cover the psychosocial needs of the affected people. Unfortunately, this historical context has often been overlooked in the transformation of services towards a recovery orientation, and thus that the concept has begun to lose its inspiration and ultimate goal, which is simply to restore people with severe mental disorders their sense of dignity, respect, self-esteem and citizenship (10). However, in recent decades, the concept of initial recovery has grown strongly in the treatment of people with mental health problems, mainly due to two pieces of scientific evidence: (a) 33% of people who show a severe mental health problem, such as schizophrenia, can recover without suffering any negative consequences and 67% show significant improvements over time; and (b) different studies have indicated that care focused on recovery (mainly, the positive expectation of having a meaningful life) predicts clinical improvement and adequate community functioning (11). In this way, in the first definitions of recovery, developed by Patricia Deegan and William Anthony, recovery implies the development of a new meaning and purpose in life, regardless of the limitations derived from the mental health problem (9).

Although this definition of recovery, due to its subjective nature, has usually been measured qualitatively, in recent years, objective instruments have emerged that assess this level of recovery (12), with an increasing number of studies that have identified the factors underlying this conceptualization. Factors, including sociodemographic (gender and age) or clinical (level of symptomatology) factors or more rehabilitative aspects, such as social functioning or cognition, would not be sufficient, although would, to a certain degree, be necessary, to achieve subjective or personal recovery, having identified the psychosocial variables that would have a main role in explaining recovery, such as empowerment, hope, quality of life, internalized stigma, perceived social support, social satisfaction, degree of recognition, loneliness and self-esteem (12, 13).

In the recovery model, the care provided by mental health professionals is characterized by a main function of supporting the affected person’s life project in such a way that they provide integrated care, aimed at promoting personal (2, 14) recovery through techniques based on the evidence of shared decision making, advance directives in mental health, the peer strategy, and training and self-care in the physical, mental and social spheres. It is a model that promotes active citizenship in the defense and is aimed at claiming the rights of people with mental illness.

### 1.2. MOSAIC: care and practices oriented towards personal recovery

MOSAIC is a social initiative, coordinated with health, that promotes the quality of life of people who suffer from mental health problems and addictions in Central Catalonia. Specifically, the project is located in Manresa, capital of the Bages region, with its own idiosyncrasy: a semi-urban area dependent on the capital in a territorially dispersed territory. It is a pioneering initiative in Catalonia, and although the project has impacted a small number of people (due to the very capacity of the services), we believe that it can promote similar experiences in Catalan territory and generate more evidence.

The Mosaic legally depends on the Tomàs Canet Foundation and is managed with the participation of four other entities: the Germanes Dominiques de Santa Clara, the Order of Sant Joan de Déu, Manresa City Council and the Althaia Foundation.In the Convent of Santa Clara, the headquarters of the project, different social and health services come together with the aim of improving people’s quality of life: (1) Work Program (WP), specialized social service that offers support and individualized advice in the search, access and maintenance of work; (2) Social Club (SC), a specialized social service that aims to increase participation and connection with the community; (3) Community Rehabilitation Service (CRS) is a specialized health service that develops different actions aimed at the psychosocial rehabilitation of people with mental disorders, which integrate the individual, group, family and community care levels to respond to their needs and personal characteristics; And (4) Individualized Service Plan (ISP) is a specialized health service that works according to an organizational model of case management and an assertive community intervention model, in order to guarantee the continuity of care and the maximum possible recovery in relation to people with a severe mental health disorders.

The fact that we can all recover does not mean that we will all do so at the same pace or following the same path (15). Mosaic adapts to the rhythm of the person. Each person must construct the meaning of his own life, he must find the resources that serve him for his well-being, he must strengthen or build an identity that is not defined by the pathology. The services are oriented towards recovery, defined as a process, that is to say, a whole set of small everyday actions that, done over time, help the person.

To better understand what Mosaic is and what its distinctive characteristics are, we can follow the US Substance Abuse and Mental Health Services Administration (SAMHSA), which proposes 10 Basic Principles of Recovery (16):

1. It comes from hope.
2. It is person-centered.
3. It occurs through many pathways.
4. It goes beyond professional care.
5. It is enriched with mutual support.
6. It assumes community.
7. It requires a comprehensive approach.
8. It is sensitive to diversity.
9. It is based on respect.
10. It requires addressing the trauma.

The last point is a very important one. Throughout our lives, people can experience painful situations that lead to a personal process that can be difficult to navigate (17, 18). A possible path towards acceptance of situations that have caused us suffering consists of facing some challenges. First of all, we need to become aware of our own experience and the possible changes that may arise in the social and relational sphere. Secondly, it is very important to have a space for the expression and management of the different emotions that can appear and overwhelm us such as sadness, anger, frustration, etc. Thirdly, we will often need to reset ourselves and not cling to the past. It is about adopting a hopeful vision of the new situation, of the present and the future, of strengthening the capacity for resilience to emerge strengthened and transformed from adversity.

### 1.3. Integrated care, a necessary challenge to address in mental health

As already mentioned, integrated care is a challenge for the world population (1–3) of which care for people with mental health problems is very present. There are two recent systematic reviews (2017 and 2022) that address the challenge. The first one (19) highlighted the efficacy demonstrated in the 172 experiences analyzed. However, it concluded with the need to obtain quality indicators, aimed at improving implementation.

In this line, Chan and his research team (20) exposed the precariousness of existing services in all health care for people with mental health problems. A very important detail of his research is the need for multidisciplinary teams with the aim of promoting transversal knowledge in the team. Finally, there is an Australian experience of integrated mental health care, designed on the personal recovery model (21). The need to promote evidence-based psychosocial interventions is highlighted, and to collaborate permanently with community organizations.

In this context, of the need to generate evidence on integration processes, in the paradigm of the personal recovery model, our study and the Mosaic project are of great importance. The need to identify quality indicators is vital to implement improvements in services and to be able to respond to a global public health problem.

### 1.4. Current context: a window of opportunity

The implementation of the perspective of recovery in the care of people with mental health problems is limited in our environment, but it is strongly considered in other countries, receiving the support of governments and public administrations, such as in the United States, New Zealand, Australia, England or Canada (22). This fact is mainly due to the fact that transformations must take place in mental health devices, and this implies that not only must the results be measured through recovery, but also that changes must be produced that promote recovery in the attitudes of professionals and in those of the people affected, so that resistance to change is softened (23–25). It is important to encourage citizen participation and orientation to the rights of people with mental illness (26).

In Catalonia, the community psychiatry resources that are using recovery-focused care characteristics, such as the Individualized Service Plan teams, use a modification of the assertive community treatment model, which provides comprehensive care (housing, socialization, symptoms, training, work, spirituality, among others); however, all Community Rehabilitation Services are prepared to provide it. On the other hand, programs, such as Activa’t per la Salut Mental, promote the personal recovery model in the social and professional fabric of the country (26, 27). The objective of this program is to (1) accompany people with mental health problems in the construction of a life project and (2) promote social support networks.

At present, in Catalonia, there is a very propitious context to promote mental health interventions using the personal recovery model: the National Pact for Mental Health (PNSM). The PNSM (28) is the interdepartmental and intersectoral instrument of the Generalitat de Catalunya that, in line with the recommendations of the World Health Organization (29), promotes mental health from all spheres of action by the government and society. Among the objectives of the PNSM, we highlight that it (1) guarantees a comprehensive, responsible and community approach, placing people and their families at the center; (2) promotes a paradigm shift in public policies related to mental health so that it is concerned with the mental health of people at different stages of life and guarantees the right of affected people to full citizenship, community inclusion and job placement; and (3) includes the conclusions of the United Nations Convention on the Rights of Persons with Disabilities (30).

This article culminates the implementation project of the personal recovery model in Central Catalonia. The result of the project was three articles aimed at promoting practice and intervention models centered on the will of the people.

## 2. Materials and methods

### 2.1. Study design

A cross-sectional non-controlled follow-up study with ex post outcomes measurements was used. This is the third study of a 5-year investigation into the recovery model. This article was preceded by a (1) systematic review and (2) mixed methods approach. In this paper we focus on a quantitative approach.

This study examined the relationship between the level of meaningful activities and other factors associated with the mental health recovery pattern. The objective this study was to assess the effectiveness of the implementation of the recovery model in a sample of people with serious mental health problems treated at MOSAIC. Our hypothesis is that the implementation of the recovery model will lead to the correlation of meaningful occupation with recovery-oriented variables. The recommendations of the Declaration of Helsinki (WMA, 2015) were followed. All persons participating in the trial signed an informed consent for their participation. This project was evaluated by the Research Ethics Committee of the participating center: Fundació Unio Catalana Hospitals, CEI 19/09.

### 2.2. Participants

The study participants were people between 18 and 65 years of age; with a diagnosis of severe mental disorder (Schizophrenia and Psychotic Disorders Cluster; Bipolar Disorder and Major Affective Disorders Cluster; Personality Disorders); no gender difference; it is linked in the 4 services of Mosaic simultaneously; and willing to participate voluntarily. Exclusion criteria: ages under 18 years and over 65 years of age; present levels of high dependency and acute destabilization of the mental health problem; language difficulties in terms of understanding and expression of the Spanish or Catalan language; presence of head trauma, dementia or severe physical disability (disabling diseases that cause a disability greater than 80%) or intellectual disability (IQ < 70); not wanting to participate in the study voluntarily. Presenting comorbidity with substance use disorders, personality disorders and organic disorders were not reasons for exclusion.

A reference professional from each device assessed the suitability to participate of the people who meet the inclusion criteria and invited them to do so. This person also facilitated the documentation of the study and had the affected person sign the informed consent. Once the person signed the informed consent, they were entered into a database, where another professional outside the study carried out the coding.

#### 2.2.1. Creation of a new structure

Integration processes require joint and coordinated work, in which professionals feel that they are part of the reorganization process. Therefore, three levels of coordination were defined in which all services were represented. (1) Driving group: Its role in the process is that of the design, start up and evaluation of the process. It is a multidisciplinary space, free for reflection, which is marked by a horizontal work dynamic. Its role, especially in the design and the first steps, was to define and mark the phases of the process. The participants in this group were: coordination project; WP; SC; CRS and ISP. Monthly meetings were held (2) Case management: Space in which all the referrals that reach MOSAIC are shared. It is a place where the first interview is reflected on based on the needs detected by the colleagues at the mental health center. It is a coordination space in which all the professionals who can potentially accompany the person are present: from professionals from the mental health center to colleagues from the social club. The frequency of the meetings was weekly. (3) Activities Commission: Place from where the joint activities of MOSAIC are designed. It follows the same multidisciplinary dynamic and the objective is to offer a range of occupations to the person from a broad perspective of recovery: health and healthy habits; work and active life; community; functioning. The existence of an internal management commission is responsible for optimizing communication channels. A periodicity of bimonthly meetings was maintained.

### 2.3. Intervention

The MOSAIC intervention is an example of support for people in the construction of their life project. The project’s strategy focuses on comprehensive and integrated care, which revolves around a single entry mechanism for 4 services. Below we detail the actions of each service and how they integrate with each other.

– Work program. Resources that are available to users with the will and ability to work. It is important for job placement and training. The labor technical offices work to reduce the obstacles that hinder the insertion and permanence in the labor market of people diagnosed with a mental health disorder. The work methodology is based on individualized monitoring and support, and group work actions.
– Social Club. Self-managed voluntary resources for users with commissions, where different activities (workshops, outings …) are carried out according to their own will to promote social and community inclusion. The word “club” refers to a group of people who are organized collectively with common rules and objectives (sporting, recreational, cultural, etc.) in relation to shared hobbies. In the case of clubs for people with mental illness, there is a care aspect that is shaped by the work of a professional team and the rehabilitative orientation of their activity. The social club service is a program of support for integration and community insertion through leisure aimed at people with mental illness in a situation of dependency. It is based on the creation and stimulation of relational links to improve the sense of belonging of the collective in the fight against social stigmatization.
– Community Rehabilitation Service. The community rehabilitation service is a public and free community rehabilitation service that offers care to older people with severe mental disorders where the personal, family and social rehabilitation and normalization of the user is worked on. The service is that therapeutic space, located at the heart of the community, which allows the user to remain integrated in their environment. It is aimed at the psychosocial rehabilitation of those people with a certain degree of autonomy and stability, and who do not present situations of acute decompensation. Different areas of the person served are worked on: social skills; the body; cognitive skills; occupational skills; the organization of leisure and free time; work skills; the family sphere; health education. Individualized service plan.
– Individualized care program aimed at the community of people who have a severe mental disorder. The professionals who make up these teams help the user connect to the health and social services he needs in his place of residence. Thus, the affected person learns to use existing resources of all kinds in an appropriate way to have their needs covered. Each professional will take about fifteen cases and will do so until the person has their deficiencies resolved.

In addition, a training program was designed based on the principles of the recovery model (11), specifically including recovery education training sessions (31, 32): emotional and material well-being of the participants; skills for the search and maintenance of meaningful occupations: work and leisure; promotion of social support networks and the care environment; and perspective of rights in the exercise of their citizenship (see Table 1).

**Table 1:** Education training sessions.

Health	
Emotional well-being	Material well-being
Satisfaction	Housing
Self-concept	Work placement
Absence of stress	Income
	Physical well-being
	Health care
	Sleep
	Health and its alterations
	Activities of daily life
Work and active life	
Personal development	Leisure
Limitations/capabilities	Relational
Access to new technologies	Cultural
Learning opportunities	Digital play
Work skills	
Functional skills	
Community	
Interpersonal relationships	Social inclusion
Social relations	Integration
Have clearly identified friends	Participation
Positive social relationships	Accessibility
Partner relationships and sexuality	Support
Family relationships	
Autonomous development	
Self-determination	Rights
Personal goals and preferences	Privacy
Autonomy	Knowledge and exercise of rights
Decisions	Respect
Elections	

In the recovery model, a basic premise is the participation of people with psychiatric life experience, as active agents in the process (11). At Mosaic they play an active role as facilitators of activities in the community rehabilitation service and in the social club: healthy habits, leisure and free time, culture. They are self-managed spaces where the presence of the professional is very reduced or absent and where the professional remains in the background.

#### 2.3.1. Design of a single referral process

As has been commented, the existence of a motor group centralized the design of the project. However, a horizontal work environment was generated in which all decisions were agreed upon (equal power relations). The number of participants, between 6 and 8 people, represented all MOSAIC services. The function of the group, in addition to designing and structuring the implementation phase, was in charge of ensuring the correct implementation. The group as a whole has a long experience and connection to the project, which made it easier to adhere to the new proposed changes. The team is oriented in a perspective of accompanying recovery processes based on respect for the rights of the person.

**Figure 1:**
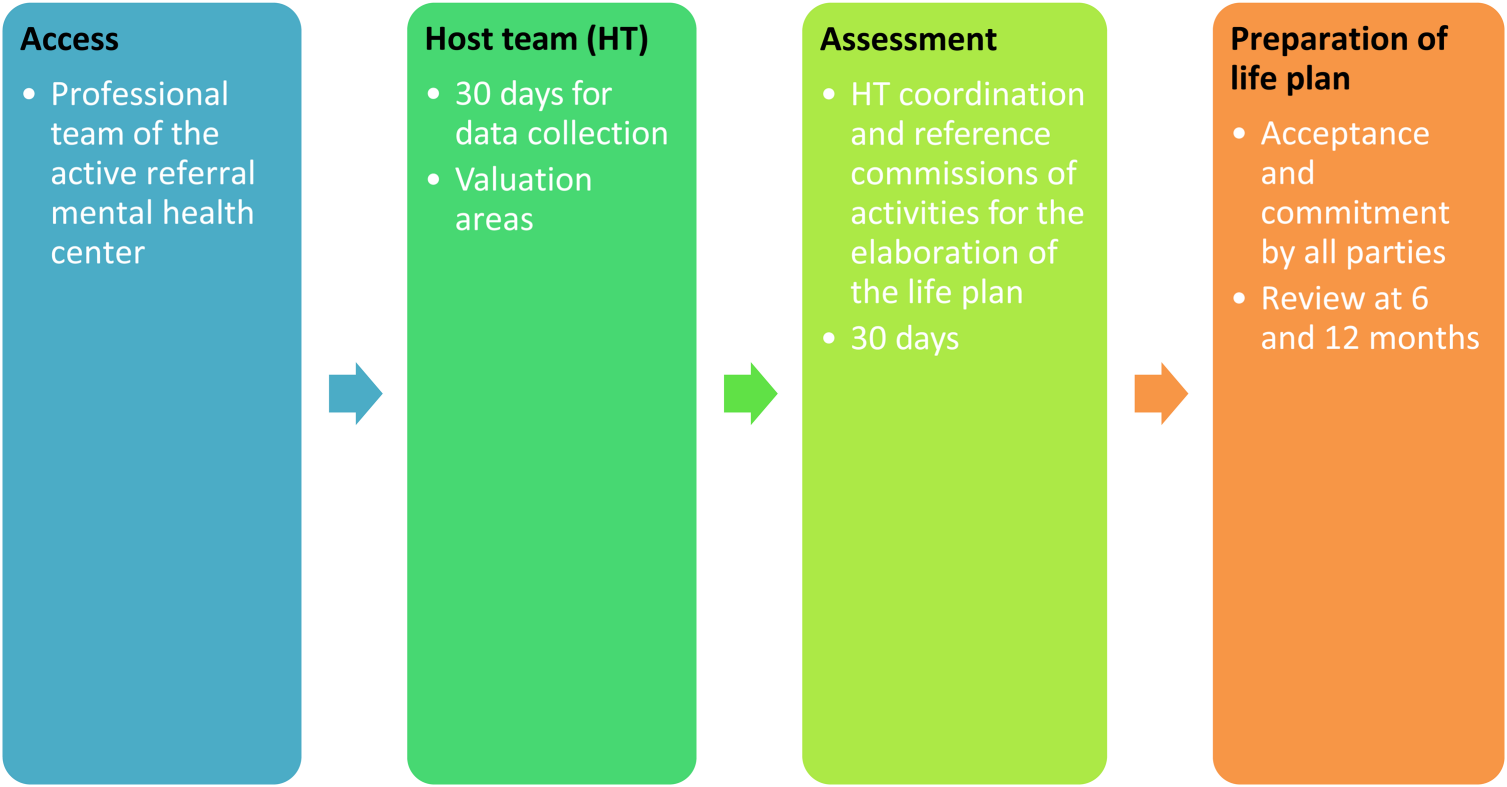
Access MOSAIC services.

Traditionally, to access MOSAIC services, mental health center professionals, mostly psychiatrists, activate a referral. With this model, it may be the case that a person has different references with the corresponding access interviews. The model we propose is to create a single referral channel to MOSAIC, reflecting personal needs (Figure 1). To achieve this, a new referral sheet (single) was designed in which the needs of the person in all their personal and social spheres were reflected. Once the referral reaches MOSAIC, it is the professionals who, based on a motivational interview, explore the person’s needs. It will be at this time when, by mutual agreement, the inclusion of the person in one of the programs becomes effective. A single database is created, accessible to all workers, where the singularities of the person and their environment are widely collected (see Supplementary Material S1).

### 2.4. Outcome variables and measures

The objectives of this study were: Engagement in Meaningful Activities Survey; The Connor–Davidson resilience scale; Hope; Recovery. The measuring instruments were selected according to variables of the recovery model. A fact of great significance is that all instruments are self-applied.

(1) The Engagement in Meaningful Activities Survey (EMAS) (Cronbach’s *α* = 0.91) reflects multiple proposals for occupational therapy and occupational science that address constituents of meaningful engagement. The EMAS addresses the assessment of the meaning of an occupation by bringing together diverse viewpoints on meaning and employment (33).
(2) The Connor–Davidson Resilience Scale (CD-RISC) (34) consists of 25 items with a Likert-type response format with five response options (“not at all”, “rarely”, “sometimes”, “often” and “almost always”), scored from 0 (“not at all”) to 4 (“almost always”). The Spanish version of the 10-item CD-RISC has a Cronbach α coefficient of 0.85 and the test–retest intraclass correlation coefficient of 0.71 (35).
(3) General Self-Efficacy Scale (GAS) (Schwarzer, 1993), in the Baessler and Schwarzer (1996) version. It consists of 10 items with responses on Likert-type scales of 5 points between 1 (totally disagree) to 5 (totally agree). Scores between 27 and 38 points show an average of general self-efficacy. This is reliable with values of *α* = .87 for the Spanish version (36).
(4) The Herth Hope Scale (37) was designed to measure goal-directed thinking across different situations. It is composed of 12 items that measure pathways and agency components by means of 4 items each, and 4 more filler items are added. In the validation studies, it had a high internal consistency (*α* = .97) and adequate divergent validity with hopelessness of −.77 (38).
(5) The Recovery Assessment Scale-revised (RAS-R) (Cronbach’s alpha ranging between *α* = 0.93 and *ω* = 0.95) is a self-applied instrument that measures personal recovery, developed over 20 years ago by Gifford and colleagues in the United States. The RAS-R consists of 24 items on a five-level scale “strongly disagree,” “disagree,” “not sure,” “agree,” and “strongly agree” (39).

### 2.5. Data collection procedures

Over the course of 12 months (September 2019–June 2020) a cross-sectional sample was identified by professionals, with prior training to unify data collection criteria, from all the participants included in the study. The team of researchers explained the research project to all the participants of Mosaic, a meeting where the inclusion criteria and the different phases of the research were detailed. Next, the reference professionals will explain again only to those people who met the inclusion criteria. Finally, their consent was collected in case of expressing a will to continue with the investigation. The reference professionals explained each of the measures (self-applied) to the study participants, giving them the opportunity to fill them in at home. Once the study participants had the measurements, the reference professional followed closely, where he accompanied the person in any doubts. Once the scales were completed, they handed them to their referral professional. The measures were shielded in order to maintain the anonymity of the responses. Finally, these were delivered to the research team and were entered into the database.

### 2.6. Data analysis

Two researchers (G.P. and I.C.) used the SPSS software (version 28.0). Pearson correlation tests were carried out to study the relationship between significant employment and the different factors using the statistical package SPSS/PC + (v. 28.0). Bonferroni correction was used to adjust the alpha to the multiple correlations.

## 3. Results

### 3.1. Demographic characteristics of the participants

The basic characteristics of the participants are shown in Table 2. A total of 59 participants were included, with a mean age of 49.0 ± 11.0 years. Of these, 47% were women, 67.3% were single, 42% had a diagnosis of psychosis, 60% had a basic level of education and 40% received income from disability benefits. Despite having a small sample, responses were collected from 80% of people who met inclusion criteria.

**Table 2:** Sociodemographic and clinical profile.

Characteristics	Participants (*N* = 59)
Age (mean, SD)	49.0 (±11.0)
Gender (% women)	47
Diagnosis (% psychosis)	42
Civil status (% single)	67.3
Family unit (% alone)	36
Income (% disability benefit)	40
Education (% basic)	60

### 3.2. Correlation analysis

The correlation matrix for the key variables is presented in Table 3.

**Table 3:** Descriptive statistics and correlations among the key variables (*n* = 59).

Variable	Mean (SD)	1	2	3	4	5	6	7	8	9
1. Self-efficacy	24.25 (4.53)		0.349*	0.222	0.437*	0.480**	0.228	0.384**	0.396**	0.152
2. Meaningful activities	39.76 (7.03)	0.112*		0.414**	0.489**	0.400**	0.368**	0.360**	0.415**	0.385**
3. Personal recovery	76.90 (13.50)	0.117	0.014**		0.465**	0.439**	0.231	0.294*	0.272	0.438**
4. Hope	21.21 (3.53)	0.480**	0.400**	0.439**	0.440**		0.082	0.333*	0.126	0.106
5. Resilience	50.55 (11.71)	0.384**	0.360**	0.294*	0.419**	0.333*	0.407**		0.054	0.189

SD, standard desviation.

* Correlation is significant at the 0.05 level.

** Correlation is significant at the 0.01 level.

The scores obtained from EMAS reflect a perception of the meaning of their activities as moderate (39.76). Meaningful activities was significantly correlated with self-efficacy (*r* = 0,112, *p* < 0,05); recovery (*r* = 0,414, *p* < 0,01); hope (*r* = 0,400, *p* < 0,01); resilience (*r* = 0,360, *p* < 0,01).

On the other hand, the GSE results place the perception of self-efficacy at an intermediate point (24.25), on a scale from 10 to 40, which indicates more self-efficacy. Self-Efficacy was significantly correlated with meaningful activities (*r* = 0,349, *p* < 0,01); empowerment (*r* = 0,437, *p* < 0,05); hope (*r* = 0,480, *p* < 0,01); resilience (*r* = 0,384, *p* < 0,01); and self-stigma (*r* = 0,396, *p* < 0,01).

Continuing with the description of the results, a RASR score of 76.90 is observed. Recovery was significantly correlated with meaningful activities (*r* = 0,014, *p* < 0,01); hope (*r* = 0,439, *p* < 0,01); resilience (*r* = 0,294, *p* < 0,05.

Continuing with the analysis, HHS stood at a score of 21.21 out of 48, with higher scores indicating greater hopefulness. Hope was significantly correlated with self-efficacy (*r* = 0,480, *p* < 0,01); meaningful activities (*r* = 0,400, *p* < 0,01); recovery (*r* = 0,439, *p* < 0,01); resilience (*r* = 0,333, *p* < 0,01).

Finally, a CD-RISC score of 50.55 out of 100 can be observed, with higher scores corresponding to higher levels of resilience. Resilience was significantly correlated with self-efficacy (*r* = 0,384, *p* < 0,01); meaningful activities (*r* = 0,360, *p* < 0,01); recovery (*r* = 0,294, *p* < 0,01); and hope (*r* = 0,333, *p* < 0,05).

After applying the Bonferroni correction (*p* < 0,005), significant positive relationships were observed between meaningful employment and the personal recovery scale (*p* = 0,003); hope (*p* = 0,004); life satisfaction (*p* = 0,002); perceived social support (*p* = 0,005); and empowerment (*p* = 0,001). The correlation coefficients with meaningful activities are presented in Table 4.

**Table 4:** Correlation coefficients with meaningful activities (*n* = 59).

Variable	*r*	*p*	*B*	*t*	95% CI
1. Self-efficacy	0.349	0.012	−0.012	0.134	(0.049, 0.584)
2. Personal recovery	0.414	0.003*	−0.010	0.128	(0.140, 0.628)
3. Hope	0.400	0.004*	−0.001	0.109	(0.175, 0.605)
4. Resilience	0.360	0.009	−0.011	0.150	(0.038, 0.605)

R, Pearson correlation; B, Bias; t, standard error; CI, confidence interval.

* Correlation is significant at the 0.005 level.

## 4. Discussion

The work addresses a topic of special relevance in the context of Catalonia (28), given a problem of global interest: the care of the person in an integral and holistic way (1–3, 19, 20). The document has identified quality indicators aimed at personal recovery (21). We believe that despite the small sample in which the project has impacted, the study facilitates the promotion of health and social integration experiences. Especially in semi-urban and rural environments, which are the usual norm except for the metropolitan area of Barcelona.

Our article contributes to the construction of evidence and to consolidate the paradigm of personal recovery in comprehensive care in Catalonia. There is evidence of the recovery model that is in tune with the results obtained (40, 41). People in recovery must be involved in all aspects and phases of the process. Thus, recovery-oriented care is characterized by:

(1) Contemplating the promotion of a positive self-concept and identity;
(2) The development of a life project beyond the mental health problem;
(3) With the hope of being able to carry it out;
(4) The promotion of self-responsibility regarding both the life project and its therapeutic process;
(5) Facilitating the creation of support and a social network;
(6) Providing tools and fostering skills to manage the disease; and
(7) Increase resilience to stressful life situations and the stigma associated with the disease (42).

All human beings are occupational beings who interact in an environment. One of the objectives of all humans is to develop occupations that are interesting to us and afford value to our existence. The results obtained in this study are in line with those of Meyer (precursor of occupational therapy), who noted the need to accompany the person through meaningful occupations (43). Contemporary authors, such as Simó-Algado and Guzmán (44), have emphasized the need to weave a life project through meaningful occupations.

### 4.1. In search of meaningful activities

Hope in moments of fragility is a transformative mechanism that promotes change and recovery, and is a pillar of the personal recovery model. Many individuals with mental health problems show confusion in the initial phases, families lack tools and the associated stigma in our communities has an impact on the recovery process (45).

Studies such as the one by Nuslang commented on the need to incorporate hope as a central element of the intervention (46), and in the pilot peer to peer test, the participants’ narratives highlighted the importance of having a meaningful occupation (47).

However, how do we promote it in our services? In the study by Hayes (48), the levels of hope between the community population and people with mental health problems were compared, obtaining significantly lower results in the study group. The conclusions they reached is that it is difficult to foster hope if the person with mental health problems has serious symptoms.

Next, we reflected on how we can generate a feeling of hope in people. As we observed in our systematic review, mutual support networks, sharing with an equal weight, are a cross element (49). MOSAIC promotes an occupational environment in which to share and forge bonds and increase social support networks. Another important aspect that the research has shown is the impact of meaningful occupations on the perception of quality of life and resilience of the participants. Both aspects have a great impact on the recovery process and are interrelated. In a study carried out in Canada (50), a direct relationship was observed: the higher the quality of life, the higher the levels of resilience. In addition, Hadebe and Ramakumba noted the importance of social networks, which influence a greater resilience in people (51).

Participation in meaningful occupations affords meaning to the recovery process and promotes resilience strategies in the face of a possible traumatic situation (52). Additionally, we found studies that support the results regarding the existing correlation between EMAS and the perception of self-efficacy (53).

This is fully consistent with the MOSAIC project and the need to establish integration processes between health and social services. It is also an opportunity to generate multidisciplinary projects and interventions, with professionals who carry in their essence a critical reflection on their praxis. These new figures are essential, as we showed, to promote an atmosphere of hope in the recovery process (54).

In addition, the construction of a meaningful life process based on meaningful occupations is key, not only because it gives hope (the basis of recovery), but also because it is essential for the person to develop full citizenship and contribute to their society as a citizen of law (55). The exercise of citizenship entails the freedom to participate in society and to be able to decide one’s life. A dignified life for each person and that corresponds to the possibilities of personal fulfillment and access to opportunities to live in health. It is a process of construction of identity and belonging (13, 26, 29).

A transcendental factor to promote full citizenship, and personal recovery, is to co-create together with the community. Studies such as the systematic review of Chan et al. (20) strongly recommend generating synergies with community assets. For this, it is essential to co-create community mental health interventions (26, 27) with the objective that people become health assets. Participating in the community and having a meaningful life project is a human right (25, 30).

### 4.2. Limitations

The present study has, of course, some limitations. The first of these is the small sample size. Mosaic’s target population is a small *n* (compared to the studies cited), and the number of participants with inclusion criteria is low. This is related to the reference population of the different services that participate in Mosaic. Another situation that marked (and surely conditioned the study) was the COVID19 pandemic. The data collection process was inactive for a few weeks due to the impossibility of monitoring. This means that not all participants are in the same recovery process.

### 4.3. Recommendations for practice and research

In the midst of a debate on the reformulation of the mental health care system for citizens, this study shows a case of success in the territory. The results obtained are a weighty argument to replicate and generate more integration experiences. The potentiality of relevant activities (significant occupations) with the personal recovery process indicates the need to plan interventions from a holistic and comprehensive perspective.

Future research needs to quantify the impact of the intervention on the outcomes described. The project lays the foundations for an RCT, which will make it possible to build evidence around integration processes from a perspective of personal recovery in mental health. RCTs of mixed methods are recommended that allow the triangulation of the results and a better understanding of the reality analyzed. Finally, it is crucial to incorporate the perspective of territorial equity and propose projects in urban areas with high population density.

## 5. Conclusions

These data indicate that the number of meaningful activities is strongly associated with variables related to the process of personal recovery from mental health problems. Subsequent studies should determine the functional weight of these variables in the performance of significant occupations.

The integration process of MOSAIC confirms the need to accompany the recovery processes through significant occupations. Variables, such as hope and resilience, are pillars in the personal recovery model, both closely related to the performance of meaningful occupations.

Finally, we highlighted the processes of social and health integration as an opportunity to include professionals with a critical vision (occupational therapists and social workers) and complement the prevailing clinical view of the health system.

The study has connected significant occupation as a human right to exercise full citizenship, in which hope is the pillar of personal recovery.

## Data Availability

The raw data supporting the conclusions of this article will be made available by the authors, without undue reservation.
