# Supplementary material for: MOSAIC, an example of comprehensive and integrated social and health care: care and practices oriented towards personal recovery
Source: Front Health Serv. 2023 Aug 4;3:1174594. doi: 10.3389/frhs.2023.1174594 (PMC10437109; doi:10.3389/frhs.2023.1174594)
Supplement: Supplementary file 1 [file Datasheet1.docx]

| 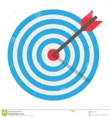**What do I want to achieve?**  Agreed objectives   \|  \| 0 – 1 – 2 – 3 - 4 \| K – R - D \| \| --- \| --- \| --- \| \|  \| 0 – 1 – 2 – 3 - 4 \| K – R - D \| \|  \| 0 – 1 – 2 – 3 - 4 \| K – R - D \| \|  \| 0 – 1 – 2 – 3 - 4 \| K – R - D \| \|  \| 0 – 1 – 2 – 3 - 4 \| K – R - D \| | | **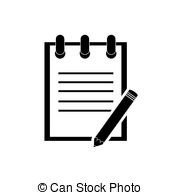What should I do?** | |
| --- | --- | --- | --- | --- | --- | --- | --- | --- | --- | --- | --- | --- | --- | --- | --- | --- | --- | --- |
| 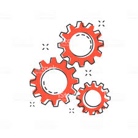**How do we do it?**  Activities | 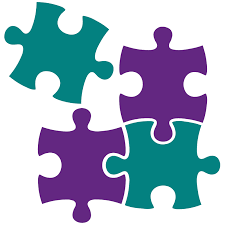**Who helps me?**  Agents involved | | **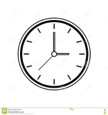When we do** |
|  |  |  | **How do I know I've done it?**  Indicators  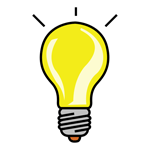 |

Instructions for the evaluation of the objectives

| 0 | 0% | not started |
| --- | --- | --- |
| 1 | 25% | initiation of the proposed actions but no continuation |
| 2 | 50% | completion of half of the proposed tasks but no continuation |
| 3 | 75% | performance of all tasks but the action of a third party is missing for their execution |
| 4 | 100% | finished |

| Keep | K | The objective in the work plan is maintained even though a) the objective has been completed 100% but it is important for the well-being of the person to influence it; b) has not been completed but it is considered necessary to continue working for the person's well-being (goal within the person's reach) |
| --- | --- | --- |
| Redefine | R | It is considered necessary to continue working towards this goal but modifications must be made to adjust to personal priorities/needs |
| Dismiss | D | The goal is dismissed either for a) 100% execution and its continuity is NOT necessary, b) goal beyond the reach of the person, c) change of personal priority |
